# *Cohnella cholangitidis* sp. nov., a novel species of the genus *Cohnella* isolated from a clinical specimen in Korea

**DOI:** 10.1007/s00203-021-02565-3

**Published:** 2021-09-25

**Authors:** Joon Ki Kim, Chi-Hwan Choi, Dae-Won Kim, Su Yeon Kim, Kyu Jam Hwang, Woo-Kon Lee, Min Kyoung Shin, Myunghwan Jung, Young Sill Choi

**Affiliations:** 1 Division of Pathogen Resource Management, Center for Public Vaccine Development Support, National Institute of Health, Korea Disease Control and Prevention Agency (KDCA), Cheongju, Republic of Korea; 2 Division of Bacterial Diseases, Bureau of Infectious Diseases Diagnosis Control, Korea Disease Control and Prevention Agency (KDCA), Cheongju, Republic of Korea; 3 Department of Microbiology, School of Medicine, Gyeongsang National University, Jinju, Republic of Korea; 4 Present Address: Division of High-Risk Pathogens, Korea Disease Control and Prevention Agency (KDCA), Cheongju, Republic of Korea

**Keywords:** *Cohnella cholangitidis*, Novel species, Complete genome, Human blood, Taxonomy

## Abstract

A Gram-positive, aerobic, rod-shaped bacterium, designated as strain 1605-214^T^, was isolated from the blood sample of a patient with cholangitis. Based on its 16S rRNA gene sequence, the strain 1605-214^T^ belonged to the genus *Cohnella* and exhibited 97.9% sequence identity with *Cohnella luojiensis* DSM 24270^T^ (GQ214052). DNA–DNA hybridization, digital DNA–DNA hybridization, and average nucleotide identity values between the two species were 23% ± 1.9, 21.1%, and 77.2%, respectively. The cellular fatty acids of strain 1605-214^T^ were mainly comprised of anteiso-C_15:0_ (36.1%), iso-C_16:0_ (16.5%), and C_16:0_ (15.1%). The predominant quinone was menaquinone-7; predominant polar lipids were diphosphatidylglycerol, phosphatidylethanolamine, and aminophospholipid-1. The cell wall peptidoglycan of strain 1605-214^T^ contained meso-diaminopimelic acid. DNA G + C content of strain 1605-214^T^ was 50.6 mol%. 5187 genes out of a total of 5413 (94.6%) were assigned putative functions using eggNOG v5.0. Based on genotypic characteristics and genomic sequence analysis results, strain 1605-214^T^ was confirmed to represent a novel species of genus *Cohnella*, for which the name *Cohnella cholangitidis* sp. nov., was proposed.

**Supplementary Information:**

The online version contains supplementary material available at 10.1007/s00203-021-02565-3.

## Introduction

The first species of the genus *Cohnella* was described as *Cohnella thermotolerans* in a report by Kämpfer et al (2006). Currently, the genus *Cohnella* comprises 37 species (LPSN: http://www.bacterio.net), including six that have not been validated. Most members of *Cohnella* were isolated from various environments such as soil (Cai et al. 2010; Kim et al. 2010, 2011), plants (Garcia-Fraile et al. 2008), water (Shiratori et al. 2010), and industrial materials (Kämpfer et al. 2006). *Cohnella cellulosilytica* (Khianngam et al. 2012) and *Cohnella faecalis* (Zhu et al. 2019) were isolated from animal excrements. *Cohnella hongkongensis* (Kämpfer et al. 2006) and *Cohnella massiliensis* (Abou Abdallah et al. 2019) were isolated from clinical samples (Table S1). In the present study, we have described strain 1605-214^T^ as a novel species of the genus *Cohnella*. To our knowledge, this is the first case in which a strain has been isolated from the blood culture of a cholangitis patient in South Korea.

## Materials and methods

### Strain isolation and identification

Strain 1605-214^T^ was isolated from the blood culture of a cholangitis patient at Gyeongsang National University Hospital in Jinju, Gyeongsangnam-do, South Korea (35°10′ 35.5′′ N, 128°05′ 44.2′′ E). The strain was grown on a blood agar plate (BAP) (KisanBio, Korea) at 30 °C for 48 h and stored at − 70 °C in 10% glycerol. Initial attempts of identification were made using matrix-assisted laser desorption/ionization–time-of-flight mass spectrometry (MALDI-TOF MS) with MALDI Biotyper software (Bruker Daltonik, Germany). The experiment was performed using *C. luojiensis* DSM 24270^T^, *C. suwonensis* DSM 25950^T^, and *C. yongneupensis* DSM 18998^T^ as reference strains for comparative analysis of species characteristics.

The 16S rRNA gene sequence similarity was calculated by comparing its sequence with those on the EzTaxon server (http://www.eztaxon.org/) (Chun et al. 2007). 16S rRNA gene sequencing was performed using universal primers 27F (3′ –AGAGTTTGATCMTGGCTCAG- 5′) and 1492R (5′ –TACGGYTACCTTGTTACGACTT-3′) (Lane 1991).

### Phenotypic, morphological, and biochemical characterization

Gram staining was performed using Gram Stain Kits (BD), and a catalase test was performed by adding 3% hydrogen peroxide solution to bacteria smeared on slides. The growth conditions for strain 1605-214^T^ were determined at different pH values (4–10, at pH intervals of 0.5 unit) on BAP. For analysis of its biochemical and enzymatic characteristics, VITEK 2 GP (bioMérieux, France) was used according to the manufacturer’s instructions.

To analyze its isoprenoid quinones, the cell biomass of strain 1605-214^T^ was obtained from cultures grown on BAP for 2 days at 30 °C. Quinones were extracted using the chloroform/methanol method [(C:M, 2:1, v/v)]. The extracted quinones were vacuum-evaporated and re-extracted using n-hexane-water (1:1, v/v). The purified quinones were analyzed using a reverse-phase HPLC system (Younglin, Korea), as described by Hiraishi et al. (1992).

The polar lipid composition of strain 1605-214^T^ was determined as described previously (Minnikin et al. 1980). The polar lipid composition was analyzed by two-dimensional thin-layer chromatography (2D-TLC) on TLC Kiesel gel 60F254 (Merck, Germany) plates (10 × 10 cm).

The cellular fatty acid composition of the isolated strain was analyzed according to Miller's method (Miller 1982). Agilent Technologies 6890 Gas Chromatography was performed to analyze the prepared samples, and an A30 m × 0.320 mm × 0.25 μm crosslinked methyl siloxane column (HP-1) was used as a separation column. The profile was analyzed using Sherlock MIS Software. Peak identification, retention time, peak area, and area ratio were determined by comparison with the standard calibration solution.

The diaminopimelic acid in the cell wall was analyzed using a previously described method (Hasegawa et al. 1983).

DNA–DNA hybridization was performed using the fluorometric microwell method (Ezaki et al. 1989).

### Genomic DNA preparation and genome sequencing

Genomic DNA was extracted by digestion of the bacteria with proteinase K in 10% SDS, followed by purification using the phenol extraction and ethanol precipitation methods. The primary sequencing library was prepared according to the protocol of the SMRTbell Template Prep Kit 1.0 (Pacific Biosciences, USA). The secondary sequencing library was prepared according to the protocol of the Ion Xpress Plus Fragment Library kit (Thermo Fisher Scientific, USA). The genome was sequenced using PacBio RS II (Pacific Biosciences, USA) and Ion S5 (Thermo Fisher Scientific, USA) sequencing platforms.

### Genome assembly

SPAdes Genome Assembler (v3.1) was adopted for de novo assembly sequence reads generated by NGS platforms PacBio RS II and Ion S5, and produced contigs and scaffold sequences. SSPACE program was used for scaffolding contigs and scaffold sequences, and the remaining sequencing errors including gaps and low-quality region were corrected using Proovread (v2.14.0).

### Genome annotation

The genome of strain 1605-214^T^ was initially annotated using the PROKKA (Seemann 2014) software package. The NCBI Prokaryotic Genome Annotation Pipeline (PGAP) (Tatusova et al. 2016) software package was used to generate the final annotation. The predicted protein sequences were classified into functional groups in Clusters of Orthologous Groups (COG) using eggNOG 5.0 (Huerta-Cepas et al. 2019). The resistance genes and virulence factors were identified using AMRFinderPlus (Feldgarden et al. 2019) and VFdb (Liu et al. 2018), respectively.

### 16S rRNA phylogenetic tree

An initial genomic distance calculation was conducted by searching for the genetically closest strains in EzTaxon Server (Chun et al. 2007) and Type Strain Genome Server (TYGS) (Meier-Kolthoff and Göker 2019). The 16S rRNA sequences of 37 type strains belonging to the *Cohnella* genus were downloaded from the list of prokaryotic names with standing in nomenclature (LPSN) (Parte 2018). The multiple sequence alignment was processed using MAFFT (Katoh and Standley 2013). Phylogenetic trees were constructed with 1000 bootstrap replicates using the neighbor-joining (NJ) method by MEGA7 (Kumar et al. 2016) and the maximum-likelihood (ML) method by RAxML (Stamatakis 2014). Figtree software was used to visualize the trees (http://tree.bio.ed.ac.uk/software/figtree).

### Genomic sequence similarity comparison

Genomic sequence similarity comparison was conducted using the available genomes of the five closest *Cohnella* species. OrthoANI (Lee et al. 2016) and digital DNA–DNA hybridization (dDDH) (Meier-Kolthoff et al. 2013) were used to compare genome similarities. To calculate the average genomic identity of orthologous gene sequences (AGIOS) (Ramasamy et al. 2014) between genomes, the sets of orthologous proteins were first obtained using BLASTP, with the reciprocal-best-BLAST-hits (RBH) approach (minimal coverage of 50%, amino acid identity of 30%). The mean percentages of nucleotide sequence identity between the orthologous genes were then calculated.

### Strain and sequence deposition

Strain 1605-214^T^ has been deposited in two microbial culture collections: the National Culture Collection for Pathogens in South Korea (NCCP), under accession number NCCP 16833, and the German collection of microorganisms (Deutsche Sammlung von Mikroorganismen und Zellkulturen GmbH in Germany, DSMZ), under the accession number DSM 112742.

The complete genomic sequences of strain 1605-214^T^ have been deposited at DDBJ/ENA/GenBank under the accession number CP041969.

## Results and discussion

### Phylogenetic affiliation

**Fig. 1:**
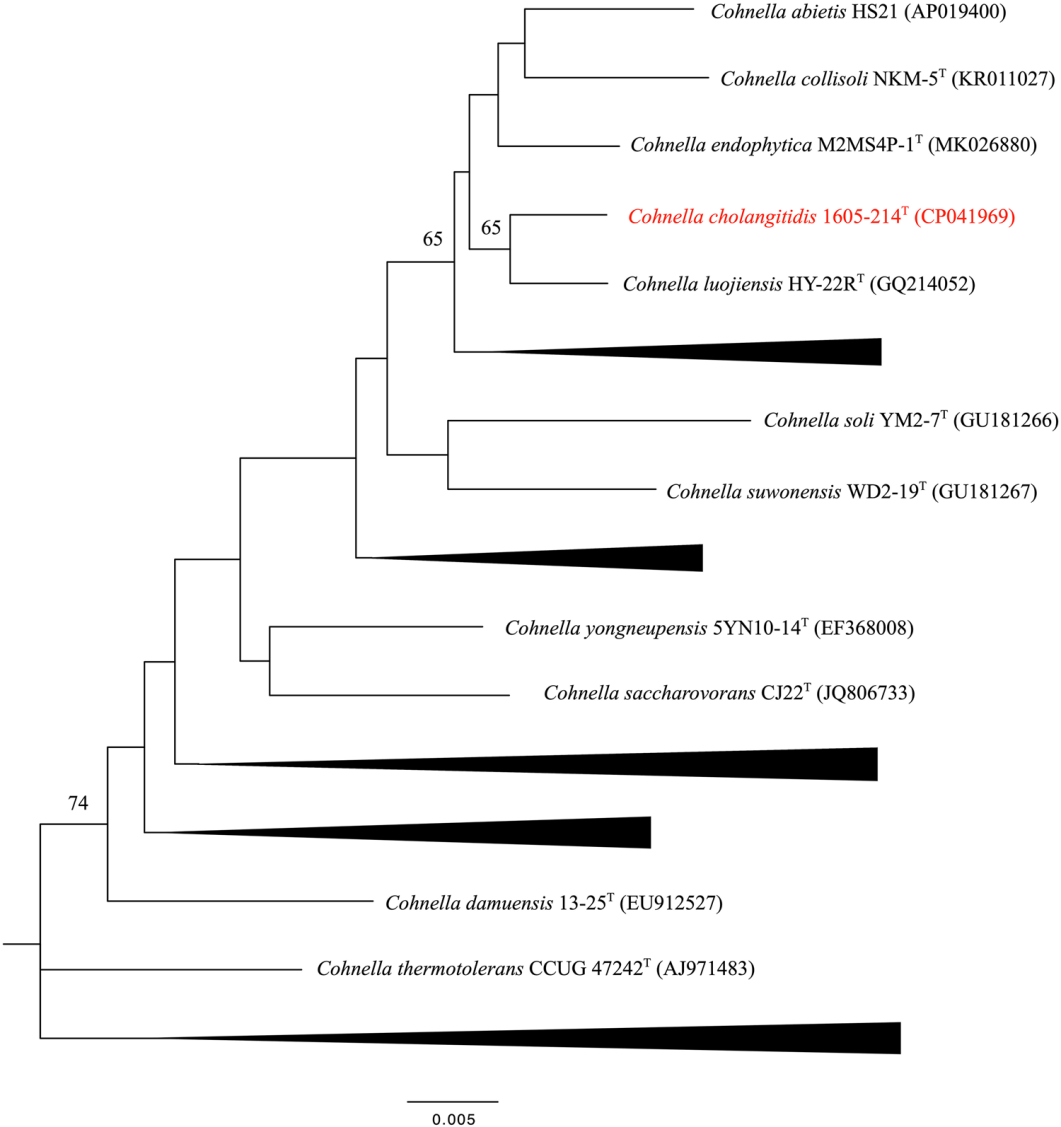
Phylogenetic tree of strain 1605-214^T^ and closely related species based on 16S rRNA gene sequences. Tree was constructed using neighbor-joining methods. Numbers at branch nodes are percentages of 1000 bootstrap replications. Only values ≥ 60% are shown. Bar under the trees indicates the nucleotide substitution rate (0.005 = 1/10E-3)

Three attempts to identify strain 1605-214^T^ by MALDI-TOF MS failed. Phylogenetic analysis, based on 16S rRNA gene sequences (Table S2) revealed that strain 1605-214^T^ belonged to the genus *Cohnella* and was closely related to *Cohnella luojiensis* DSM 24270^T^ (97.9%) (Fig. 1, Figure S1).

### Phenotype

The optimal conditions of strain 1605-214^T^ for growth were a temperature of 30 °C and a pH of 7 (Table 1). Based on VITEK 2 GP results, all four *Cohnella* spp. strains were positive for beta-galactosidase, beta galactopyranosidase, and alpha-galactosidase. In addition, strain 1605-214^T^ was positive for alpha-glucosidase and negative for D-trehalose, whereas *Cohnella luojiensis*, which is considered a genetically close species, was negative and positive, respectively, for the above-mentioned enzymes. The major lipid classes of strain 1605-214^T^ were identified as diphosphatidylglycerol (DPG), phosphatidylethanolamine (PE), and aminophospholipid-1 (APL1). The major quinone present in the strain was identified as MK-7. The cell wall peptidoglycan of strain 1605-214^T^ contained meso-diaminopimelic acid. The G + C content of strain 1605-214^T^ was 50.6 mol%, and the major fatty acids were anteiso-C_15:0_ (36.1%), iso-C_16:0_ (16.5%), and C_16:0_ (15.1%) **(**Table 2). The relatedness of DNA between strain 1605-214^T^ and *C. luojiensis* DSM 24270^T^ was 23.0% ± 1.9. Based on phenotypic and genotypic distinctness and DNA–DNA hybridization results, strain 1605-214^T^ was confirmed to be a novel pathogenic species similar to *C. luojiensis.*

**Table 1:** Comparison of characteristics of strain 1605-214^T^ and related type strains of *Cohnella* species

Characteristic	1	2	3	4
Gram stain	+	+	+	+
Catalase	+	+	+	+
Motility	−	−	−	−
Temperature range (°C)	15–42	15–37	15–37	10–37
Optimal pH for growth	6–8	6–8	6–8.5	5.5–8.5
Polar lipids	DPG, PE, APL1	DPG, PG, PE, PL1	DPG, PG, PE, APL4, APL5	DPG, PG, PE, APL2, APL3
Diaminopimelic (DAP) acid	meso-DAP	meso-DAP	meso-DAP	meso-DAP
Quinones	MK-7	MK-7	MK-7	MK-7
G + C content (mol%)	50.6	48.3	54.4	51.4
Beta-galactosidase	+	+	+	+
Alpha-glucosidase	+	−	−	−
Beta galactopyranosidase	+	+	+	+
Alpha-galactosidase	+	+	+	+
D-trehalose	−	+	−	−

All data are from the present study. +, positive; −, negative; w, weakly positive

Negative for all 4 strains: D-amygdalin, Phosphatidylinositol phospholipase C, D-xylose, Arginine dihydrolase 1, Ala-Phe-Pro arylamidase, Cyclodextrin, L-Aspartate arylamidase, Alpha-mannosidase, Phosphatase, Leucine arylamidase, L-Proline arylamidase, Beta glucuronidase, L-pyrrolidonyl-arylamidase, Beta glucuronidase, Alanine arylamidase, Tyrosine arylamidase, D-sorbitol, Polymyxin B resistance, D-galactose, D-ribose, L-Lactate alkalinization, Lactose, N-Acetyl-D-glucosamine, D-maltose, Bacitracin resistance, Novobiocin resistance, Growth In 6.5% NaCl, D-mannitol, D-mannose, Methyl-B-D-glucopyranoside, Pullulan, D-raffinose, O/129 resistance (Comp.Vibrio.), Salicin, Saccharose/Sucrose, Arginine dihydrolase 2, Optochin resistance

1—*C. cholangitidis* 1605-214^T^; 2—*C. luojiensis* DSM 24270^T^; 3—*C. suwonensis* DSM 25950^T^; 4—*C. yongneupensis* DSM 18998^T^

**Table 2:** Cellular fatty acid content (%) of strain 1605-214^T^ and *C. luojiensis* DSM 24270^T^, *C*. *suwonensis* DSM 25950^T^, and *C*. *yongneupensis* DSM 18998^T^

Fatty acid	1	2	3	4
10:00	0.1	–	–	–
12:0 iso	–	–	–	0.3
12:00	0.3	1.6	0.6	0.6
13:0 iso	0.2	–	–	–
13:0 anteiso	0.9	–	1.5	1.5
14:0 iso	2.5	2.1	4.2	2.1
14:00	1.3	1.0	1.2	1.5
15:0 iso	4.8	9.0	6	2.2
15:0 anteiso	36.1	51.2	51.9	45.1
16:1 w7c alcohol	0.5	2.1	–	–
16:00	15.1	4.9	7.7	14.0
16:0 iso	16.5	11.0	20.5	23.6
16:1 w11c	0.3	1.9	–	–
16:1 w7c/16:1 w6c	0.2	–	–	–
17:1 iso w10c	–	1.4	–	–
17:0 iso	4.2	4.3	1.9	0.9
17:0 anteiso	5.3	8.2	4.5	6.7
17:00	0.9	–	–	–
18:0 iso	0.2	–	–	-
18:00	–	–	–	0.8
18:2 w6,9c/18:0 ante	0.6	–	–	-
18:1 w9c	4.9	–	–	0.9
18:1 w7c	0.3	–	–	–
18:1 w6c	0.5	–	–	–
18:00	4.3	–	–	–
16:1 w7c/16:1 w6c	0.2	–	–	–
17:1 iso I/anteiso B	–	1.3	–	–
18:2 w6,9c/18:0 ante	0.5	–	–	–
18:1 w7c or 18:1 w6c	0.8	–	–	–

All data are from the present study. – Not detected

1—*C. cholangitidis* 1605-214^T^; 2—*C. luojiensis* DSM 24270^T^; 3—*C. suwonensis* DSM 25950^T^; 4—*C. yongneupensis* DSM 18998^T^

*Summed features represent groups of two fatty acids that could not be separated by GLC using the MIDI system

### Genome properties

The complete genome of strain 1605-214^T^ is 6,408,853 bp in length with a GC content of 51.2%. Out of the 5867 predicted genes, 5481 genes code for proteins and 95 code for RNA (8 genes are 5S rRNA genes; 8 genes are 16S rRNA; 8 genes are 23S rRNA genes; 67 genes are tRNA genes; 4 genes are ncRNAs genes) (Table S3). From the analysis of Clusters of Orthologous Groups of proteins (COGs), a total of 5,187 genes (94.6%) were assigned putative functions (Table S4). The strain 1605-214^T^ contained 896 genes (16.3%) for information storage and processing, 1224 genes (22.3%) for cellular processes and signaling, and 2108 genes (38.5%) for metabolism. An in silico search for the resistome of this strain revealed that the clbC gene (90.4% identity) (Hansen et al. 2012) confers resistance to PhLOPSa (phenicol, lincosamide, oxazolidinone, pleuromutilin, and streptogramin A) antibiotics and was identified by NCBI AMRFinder program (Feldgarden et al. 2019). An in silico search for virulence factors revealed eight proteins with high identity percentages conferring potential pathogenicity. These proteins were LPS biosynthesis protein PseA-like (79.2% identity), chaperonin GroEL (75.3%), translation elongation factor Tu (73.6%), UTP–glucose-1-phosphate uridylyltransferase gtaB (71.8%), imidazole glycerol phosphate synthase subunit HisF (71.3%), ATP-dependent Clp protease proteolytic subunit clpP (71.3%), enolase eno (70.3%), and glucose-1-phosphate thymidyl transferase rmlA (70.2%).

### Comparison with genomes of other *Cohnella* species

**Fig. 2:**
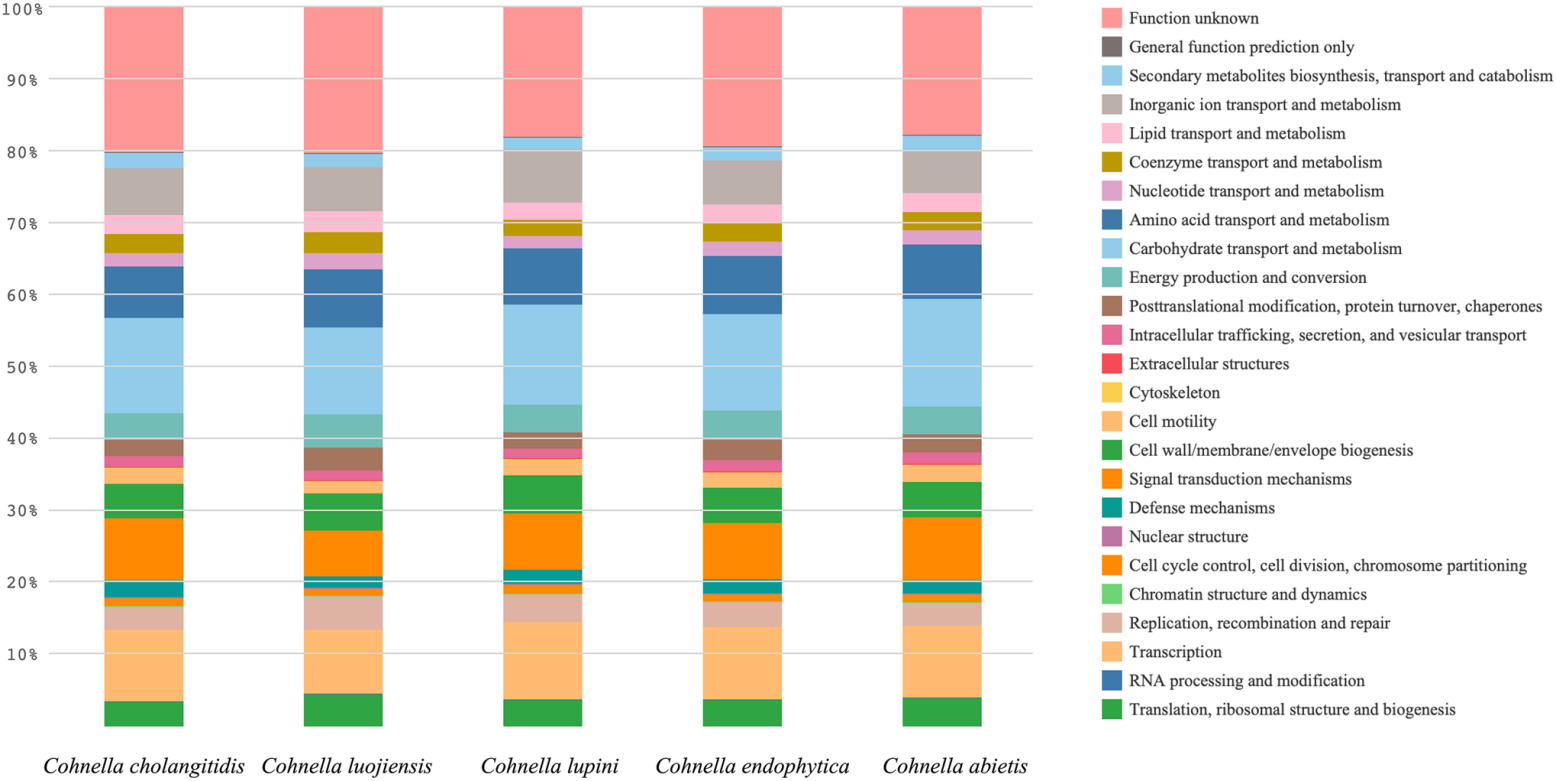
Functional COG distribution of the predicted genes in genomes. Gene components between the four closest species of genomes showed a similar composition of functional COG distribution

At the time of manuscript preparation, the 16S rRNA sequences of the type strains were analyzed as mentioned above; however, a comparison at the whole-genome level was not possible. Therefore, the *Cohnella cholangitidis* 1605-214^T^ was further compared to four type strains, including *C. luojiensis* (Table 3). The four strains were selected based on the results of the dDDH analysis from TYGS. Additionally, average nucleotide identity (ANI) analysis was also performed for the strains. The dDDH and ANI results for the assessed strains were, respectively, as follows: *C. luojiensis* (21.1%, 77.2%), *C. lupini* (20.6%, 76.1%), *C. endophytica* (20.3%, 75.4%), and *C. abietis* (19.5%, 73.7%) (Table 3). These dDDH (< 70%) and ANI (< 95%) values indicated that strain 1605-214^T^ represents a species distinct from other *Cohnella* strains. The distribution of genes into COG categories was similar in the gnomes of five strains as shown in Fig. 2. Strain 1605-214^T^ shared 3853, 4127, 4163, and 4084 orthologous genes with *C. lupijiensis, C. lupini, C. abietis, C. endophytica,* respectively (Table 4, upper diagonal numbers). The average genomic identity of orthologous gene sequence (AGIOS) ranged from 48.5% with *C. abietis* to 49.0% with *C. luojiensis* (Table 4, lower diagonal numbers).

**Table 3:** G + C content, digital DNA–DNA hybridization (dDDH), average nucleotide identity (ANI), and 16S rRNA sequence identity of the genome sequences of *C. cholangitidis* 1605–214^T^ and the four closest strains

Species	Strain	GenBank accession	Genome assembly level (contig no.)	Sequence length (Mbp)	G + C content (%)	dDDH (%)	ANI (%)	16S rRNA (%)
*C. cholangitidis*	1605-214^T^	CP041969	Complete (1)	6.4	51.2	–	–	–
*C. luojiensis*	HY-22^T^	NZ_SOMN00000000	Draft (115)	5.04	49.9	21.1	77.2	97.9
*C. abietis*	HS-21^T^	AP019400	Complete (1)	7.05	44.8	19.5	73.6	97.6
*C. endophytica*	M2MS4P-1^T^	NZ_RBZM00000000	Draft (32)	6.26	51.5	20.3	75.4	97.4
*C. lupini*	RLAHU4B^T^	NZ_QRDY00000000	Draft (65)	6.34	50.7	20.6	76.1	97.2

**Table 4:** The number of shared orthologous genes between the genomes of four closely related strains (upper diagonal numbers), the average genomic identity of orthologous gene sequences corresponding to orthologous proteins shared between the genomes (lower diagonal numbers), and number of proteins per genome (principal diagonal elements; bold numbers)

	1605-214^T^	HY-22^T^	HS-21^T^	M2MS4P-1^T^	RLAHU4B^T^
*C. cholangitidis* 1605-214^T^	5481	3853	4163	4084	4127
*C. luojiensis* HY-22^T^	48.9	4737	3979	3950	3851
*C. abietis* HS-21^T^	48.5	64.7	6203	4380	4341
*C. endophytica* M2MS4P-1^T^	48.9	66.8	64.2	5335	4199
*C. lupini* RLAHU4B^T^	49.0	71.2	64.4	67.7	5608

## Conclusion

The phenotypic, morphological, and biochemical characterizations, genome perspectives, and comparative genome analyses suggested that strain 1605-214^T^ represents a novel species of the genus *Cohnella* for which the name *C. cholangitidis* is proposed.

### Description of *Cohnella cholangitidis* sp. nov.

*Cohnella cholangitidis* (chol.an.gi’ti.dis. N.L. gen. n. *cholangitidis* of cholangitis, derived from the disease of the patient from which this strain was isolated).

Gram-positive, rod-shaped, catalase-positive, oxidase-positive, and facultative anaerobic. Colonies are grayish-white in color and 0.5 mm in size on BAP. The optimal growth conditions are 30 °C and pH 7, although growth is also observed at 15–42 °C and pH 6–8. Positive for alpha-galactosidase, beta-galactosidase, alpha-glucosidase, and beta-galactopyranosidase. The G + C content is 50.6 mol% and the major fatty acids are anteiso-C15:0 (36.1%), iso-C16:0 (16.5%), and C16:0 (15.1%). The major lipids are diphosphatidylglycerol (DPG), phosphatidylethanolamine (PE), and aminophospholipid-1 (APL1). The major quinone is MK-7. The cell wall contains meso-diaminopimelic acid.

Strain 1605-214^T^ (= NCCP 16833^T^, = DSM 112742^T^) was isolated from a clinical specimen at the Gyeongsang National University Hospital in Jinju, Gyeongsangnam-do, South Korea.

## Supplementary Information

Below is the link to the electronic supplementary material.

Supplementary Fig. S1 Phylogenetic tree of strain 1605-214T based on 16S rRNA gene sequences. Tree was constructed using maximum-likelihood method. Numbers at branch nodes are percentages of 1000 bootstrap replications. Only values ≥ 60% are shown. Bar under the trees indicates the nucleotide substitution rate (0.05 = 1/10E-2) (TIFF 10668 KB)

Supplementary file2 (XLSX 20 KB)

Supplementary file3 (XLSX 18 KB)

Supplementary file4 (DOCX 37 KB)

## Data Availability

All data generated or analyzed during this study are included in this published article and its Supplementary Information files. Additional data are available from the corresponding author upon request. Depositories: Strain 1605-214^T^ has been deposited in the National Culture Collection for Pathogens in South Korea (NCCP), and Deutsche Sammlung von Mikroorganismen und Zellkulturen GmbH (DSMZ), under the accession number NCCP 16833-DSM 112742.

## Abbreviations

BAP: Blood agar plate
ANI: Average nucleotide identity
DDH: DNA–DNA hybridization
dDDH: Digital DNA–DNA hybridization
AGIOS: Average of genomic identity of orthologous gene sequence
COG: Cluster of orthologous groups
