# Supplementary material for: Cohnella cholangitidis sp. nov., a novel species of the genus Cohnella isolated from a clinical specimen in Korea
Source: Arch Microbiol. 2021 Sep 25;203(10):6053–60. doi: 10.1007/s00203-021-02565-3 (PMC8475853; doi:10.1007/s00203-021-02565-3)
Supplement: Supplementary file 4 — Supplementary file4 (DOCX 37 KB) [file 203_2021_2565_MOESM4_ESM.docx]

**Table S3. Genomic nucleotide content and the number of genes in *C. cholangitidis* 1605-214** **^T^**

| Characteristics | Number | % |
| --- | --- | --- |
| Size (bp) | 6,408,853 | 100 |
| Number of G+C | 3,283,666 | 51.2 |
| Total genes | 5,867 | 100 |
| Protein coding genes | 5,481 | 93.4 |
| Total RNA genes | 95 | 1.6 |
| tRNA genes | 67 | 1.1 |
| rRNA (5S, 16S, 23S) genes | 24 | 0.4 |
| ncRNA | 4 | 0.1 |
| Pseudogenes | 291 | 5.0 |
| Coding sequence size | 5,761,976 | 90.0 |
| Protein coding sequence size | 5,401,037 | 84.3 |
| tRNA coding sequence size | 5,241 | 0.1 |
| rRNA coding sequence size | 37,041 | 0.6 |
| Proteins associated with COG^*^s | 5,187 | 94.6 |
| Proteins not associated with COGs | 294 | 5.4 |
| Proteins associated with unknown function of COGs ([S]) | 1,064 | 19.4 |
| Genes associated with resistance | 1 | 0.0 |
| Genes associated with virulence | 8 | 0.2 |

* Clusters of Orthologous Groups (COG)

**Table S4. Distribution of the 25 general COG functional categories**

| Class | No. families | Percentage of total | Description |
| --- | --- | --- | --- |
| [J] | 193 | 3.5% | Translation, ribosomal structure and biogenesis |
| [A] | 0 | 0.0% | RNA processing and modification |
| [K] | 521 | 9.5% | Transcription |
| [L] | 181 | 3.3% | Replication, recombination and repair |
| [B] | 1 | 0.0% | Chromatin structure and dynamics |
| [D] | 64 | 1.2% | Cell cycle control, cell division, chromosome partitioning |
| [Y] | 0 | 0.0% | Nuclear structure |
| [V] | 122 | 2.2% | Defense mechanisms |
| [T] | 457 | 8.3% | Signal transduction mechanisms |
| [M] | 253 | 4.6% | Cell wall/membrane/envelope biogenesis |
| [N] | 107 | 2.0% | Cell motility |
| [Z] | 16 | 0.3% | Cytoskeleton |
| [W] | 1 | 0.0% | Extracellular structures |
| [U] | 82 | 1.5% | Intracellular trafficking, secretion, and vesicular transport |
| [O] | 122 | 2.2% | Posttranslational modification, protein turnover, chaperones |
| [C] | 188 | 3.4% | Energy production and conversion |
| [G] | 698 | 12.7% | Carbohydrate transport and metabolism |
| [E] | 377 | 6.9% | Amino acid transport and metabolism |
| [F] | 101 | 1.8% | Nucleotide transport and metabolism |
| [H] | 142 | 2.6% | Coenzyme transport and metabolism |
| [I] | 139 | 2.5% | Lipid transport and metabolism |
| [P] | 344 | 6.3% | Inorganic ion transport and metabolism |
| [Q] | 119 | 2.2% | Secondary metabolites biosynthesis, transport and catabolism |
| [R] | 0 | 0.0% | General function prediction only |
| [S] | 1064 | 19.4% | Function unknown |
| - | 294 | 5.4% | not in COGs |

* Information storage and processing: J, A, K, L, B; Cellular processes and signaling: D, Y, V, T, M, N, Z, W, U, O; Metabolism: C, G, E, F, H, I, P, Q
